# Automated Analysis of Cell-Matrix Adhesions in 2D and 3D Environments

**DOI:** 10.1038/srep08124

**Published:** 2015-01-29

**Authors:** Joshua A. Broussard, Nicole L. Diggins, Stephen Hummel, Walter Georgescu, Vito Quaranta, Donna J. Webb

**Affiliations:** 1 Department of Biological Sciences and Vanderbilt Kennedy Center for Research on Human Development, Vanderbilt University, Nashville, Tennessee 37235; 2 Center for Cancer Systems Biology at Vanderbilt, Vanderbilt University, Nashville, Tennessee 37235; 3 Vanderbilt Institute for Integrative Biosystems Research and Education (VIBRE), Vanderbilt University, Nashville, Tennessee 37235; 4 Department of Biomedical Engineering, Vanderbilt University, Nashville, Tennessee 37235; 5 Department of Cancer Biology, Vanderbilt University, Nashville, Tennessee 37235

## Abstract

Cell-matrix adhesions are of great interest because of their contribution to numerous biological processes, including cell migration, differentiation, proliferation, survival, tissue morphogenesis, wound healing, and tumorigenesis. Adhesions are dynamic structures that are classically defined on two-dimensional (2D) substrates, though the need to analyze adhesions in more physiologic three-dimensional (3D) environments is being increasingly recognized. However, progress has been greatly hampered by the lack of available tools to analyze adhesions in 3D environments. To address this need, we have developed a platform for the automated analysis, segmentation, and tracking of adhesions (PAASTA) based on an open source MATLAB framework, CellAnimation. PAASTA enables the rapid analysis of adhesion dynamics and many other adhesion characteristics, such as lifetime, size, and location, in 3D environments and on traditional 2D substrates. We manually validate PAASTA and utilize it to quantify rate constants for adhesion assembly and disassembly as well as adhesion lifetime and size in 3D matrices. PAASTA will be a valuable tool for characterizing adhesions and for deciphering the molecular mechanisms that regulate adhesion dynamics in 3D environments.

Cell-matrix adhesions are sites of contact between a cell and the extracellular matrix (ECM) that physically link the ECM to the cytoskeleton and function to transmit extracellular signals to the interior of cells^1^,^2^,^3^. They are critical to many biological processes including cell migration, survival, proliferation, differentiation, tissue morphogenesis, tissue homeostasis, wound repair, and tumorigenesis^4^,^5^,^6^,^7^,^8^. In many of these processes, adhesions are dynamic structures that are constantly changing and remodeling. For example, adhesions must continuously assemble and disassemble, in a process termed adhesion turnover, in order for cells to migrate efficiently^9^,^10^,^11^. Adhesions are composed of a number of different proteins, including integrin transmembrane receptors, which bind to the ECM, and intracellular signaling and structural proteins, such as paxillin, vinculin, talin, and focal adhesion kinase (FAK), that link integrins to the actin cytoskeleton^12^,^13^,^14^,^15^. Many of the studies characterizing adhesions have focused on cells plated on planar 2D substrates^6^,^16^. These studies have proven to be very beneficial for identifying key adhesion proteins as well as regulatory mechanisms. However, recent work has highlighted the importance of examining adhesions in more physiologic 3D environments^17^,^18^,^19^,^20^,^21^,^22^.

Although the characterization of adhesions in 3D matrices is in its infancy, available data indicate that adhesions in 2D and 3D environments can differ, at least in some aspects^21^,^23^. For example, when fibroblasts were plated on 2D substrates or in 3D cell- or tissue-derived matrices, FAK was differentially phosphorylated in 2D and 3D adhesions^17^. Other studies have also shown differences in adhesion signaling, morphology, and composition between 2D and 3D^21^,^24^,^25^. These differences point to the need to better characterize adhesions in 3D environments. Some key proteins, such as integrins, paxillin, talin, and FAK, have been observed in adhesions in various 3D matrices^17^,^19^,^24^,^25^,^26^, which will provide useful markers for studying adhesion structure and dynamics in 3D environments.

While our current knowledge regarding adhesion dynamics in 3D environments is limited, adhesions have been shown to assemble, mature, and disassemble in cells migrating in 3D type I collagen matrices^20^. In these live-cell imaging experiments, adhesions formed along collagen fibers at the leading edge of protrusions and traveled rearward as they matured, causing fiber deformation^20^. Adhesion maturation in 3D environments has been linked to myosin II contractility and the structure of the microenvironment surrounding the adhesion^27^,^28^. Photorecovery of adhesion proteins also demonstrates that adhesions assemble and disassemble in cells migrating on one-dimensional (1D) patterned fibril-like structures, which were used as a model system for oriented 3D fibrillar matrices^27^,^29^. However, little mechanistic data for the regulation of adhesion assembly, maturation, and disassembly in 3D matrices is currently available. Progress in this rapidly emerging field has been greatly hampered by the lack of available tools to analyze adhesion dynamics in 3D environments.

To address this need, we have created an automated platform, PAASTA, for analyzing adhesion dynamics in cells migrating on both 2D substrates and in 3D environments, that is based on an open source MATLAB framework, CellAnimation^30^. We manually validate our platform using an established adhesion analysis method^9^ and use PAASTA to quantify adhesion dynamics in 3D matrices.

## Results

### An automated platform, PAASTA, for adhesion analysis

**Figure 1:**
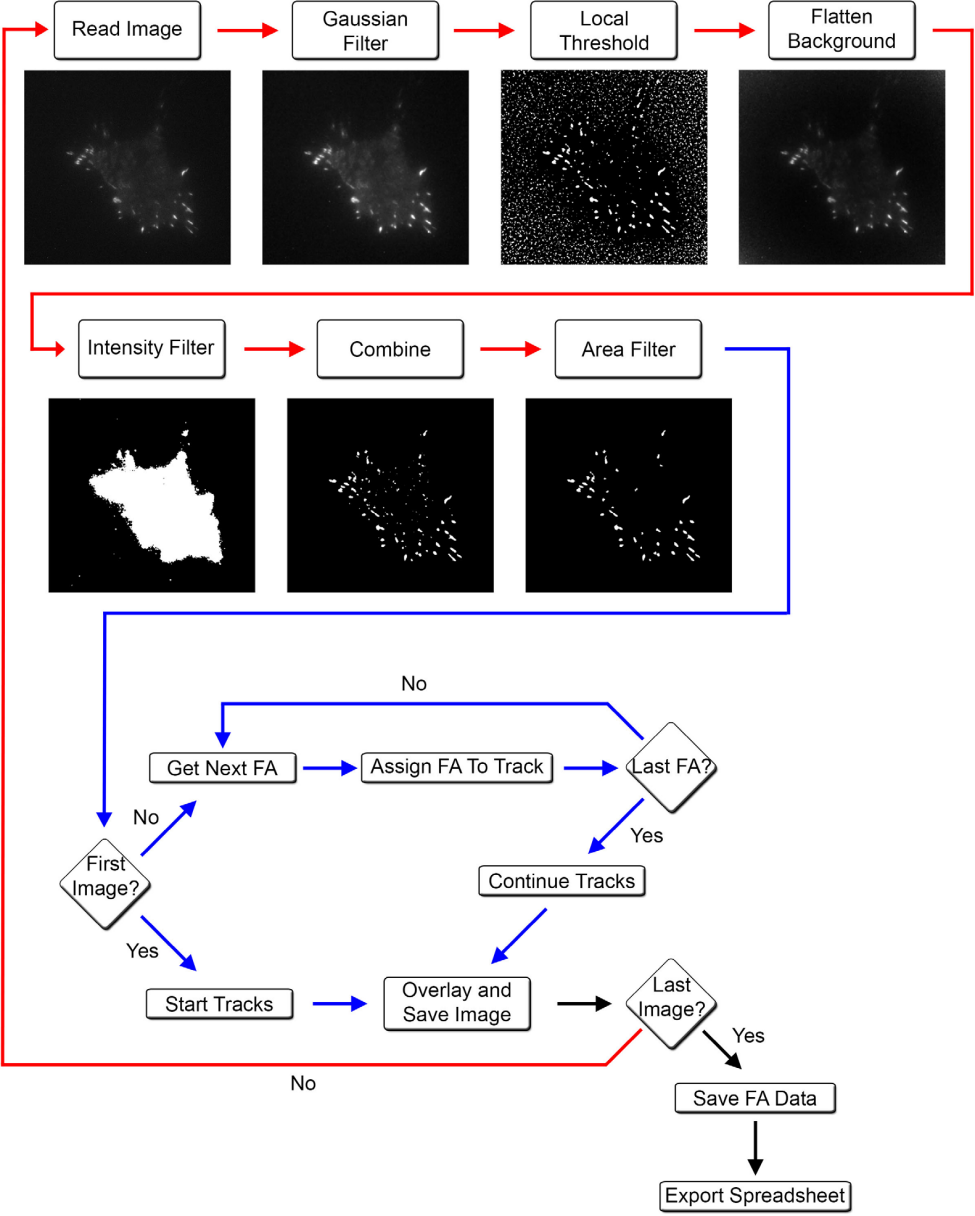
An automated platform, PAASTA, for tracking and analyzing adhesions. This platform subjects raw images to a series of steps to identify adhesions. A flow chart detailing each step with a corresponding output image is shown. A segmentation pipeline (red lines), where images are processed in order to segment adhesions, is followed by a tracking pipeline (blue lines) that identifies and tracks adhesions through a series of time-lapse images. Upon completion of the track assignment process for a particular frame, an overlay image is generated which displays the ID numbers for each adhesion in the frame. These images may be used for visual inspection of the automated tracking assignments. Intensity values are calculated for all tracked adhesions at every time point using the background-corrected intensity image. When the tracking assignment is completed for all the frames, the final adhesion ID numbers along with centroid locations and intensity values are exported to comma-separated text files for further analysis.

**Figure 2:**
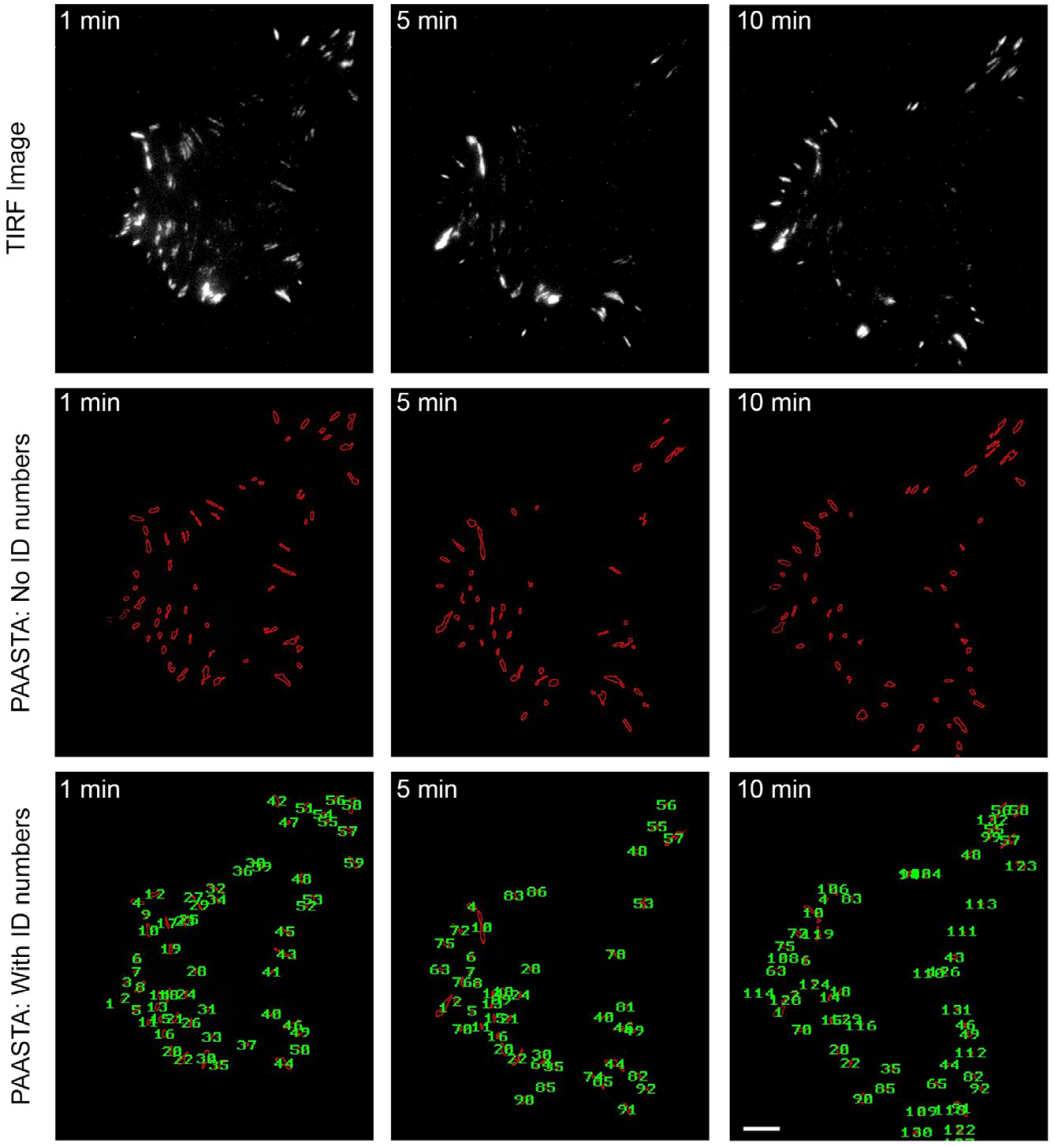
Adhesion identification and tracking using PAASTA. Raw time-lapse TIRF images of an HT1080 cell expressing GFP-paxillin are shown (upper panels). These images were then processed with PAASTA to generate individual adhesion tracks that are shown in the lower panels both without labeling (No ID numbers) and with ID numbers labeling each adhesion (With ID numbers). Bar, 5 μm.

In order to perform automated detection and quantification of adhesions over time, we begin with raw images, which are acquired with time-lapse microscopy, of cells with fluorescently-labeled adhesions (Fig. 1). Initially, a Gaussian smoothing module is applied to the raw images to reduce noise and then corrected for uneven background illumination by dividing each smoothed image with a low pass filtered version of itself. To detect adhesions, we employ a local thresholding module that compares the intensity of each pixel with the mean value of the local neighborhood of the pixel. If the value of the pixel is higher than the local average, it is classified as an adhesion pixel; otherwise, it is assigned to the background pixel class. Objects less than 1 μm^2^ are excluded to ensure that background noise is eliminated from the analysis. Cell outlines are detected by thresholding the background-corrected images using a global intensity threshold module. Adhesions are selected by combining the binary mask of the cell with the binary image of the adhesions. Individual adhesions, which are assigned identification (ID) numbers, are tracked over time using a nearest neighbor algorithm. Adhesion ID numbers, individual adhesion integrated intensities, and area information at every time point are exported to comma-separated text files for further analysis. Sets of images showing the detected adhesion outlines, with or without ID numbers, are overlaid on the original images for manual validation of the automated quantification (Fig. 2).

### Manual validation of PAASTA

**Figure 3:**
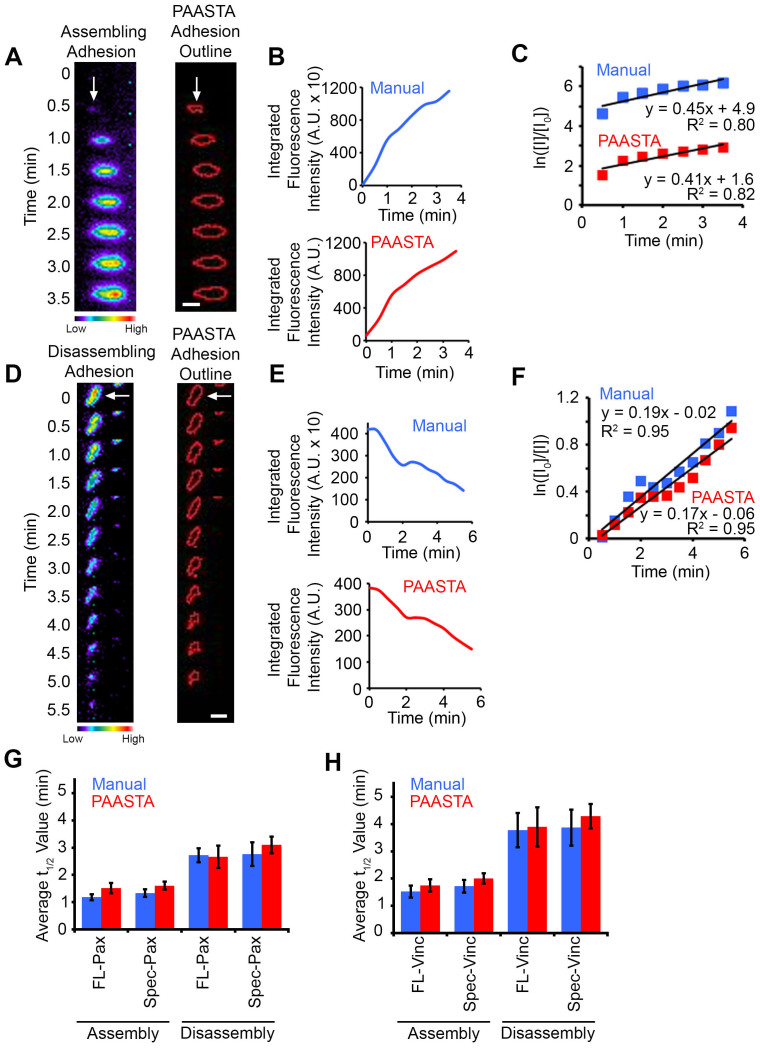
Manual validation of PAASTA with HT1080 cells. (A,D) Left, Montages of time-lapse TIRF images for an assembling (panel A) and a disassembling (panel D) adhesion (white arrows) in cells expressing GFP-paxillin are shown in pseudo-color coding. Warm colors correspond to higher fluorescence intensity values whereas cool colors represent lower fluorescence intensity values. Right, Outlines of the adhesions as segmented by PAASTA. (B,E) Graphs of the fluorescence intensities for the assembling (panel B) and disassembling (panel E) adhesion are shown for both Manual and PAASTA tracking. (C,F) Plots of the natural log of the fluorescence intensity of the adhesions at given time points (I) relative to the initial fluorescence intensity (I_0_) are shown. Trendlines with the corresponding equation (y = mx + b) and R^2^ values are shown for fluorescence intensities attained manually (Manual) and with PAASTA. The slopes of these graphs (m) are the apparent rate constant for adhesion assembly (panel C) or the rate constant for adhesion disassembly (panel F). (G) The average t_1/2_ values for adhesion assembly and disassembly for cells expressing GFP-paxillin (FL-Pax) or GFP-paxillin with a truncated CMV promoter (Spec-Pax) are shown for Manual and PAASTA tracking. S.E.M. was calculated from: 21–24 adhesions (11–14 assembly, 10 disassembly) for each construct. A total of 14 cells were analyzed for adhesion assembly and disassembly. (H) The average t_1/2_ values for adhesion assembly and disassembly for cells expressing GFP-vinculin (FL-Vinc) or GFP-vinculin with a truncated CMV promoter (Spec-Vinc) are shown for Manual and PAASTA tracking. S.E.M. was calculated from: 20–21 adhesions (10–11 assembly, 10 disassembly) for each construct. A total of 13 cells were analyzed for adhesion assembly and disassembly. A Wilcoxon rank sum test showed no statistically significant difference between Manual and PAASTA tracking for FL-Pax assembly (*Z = −1.689, p = 0.091*), disassembly (*Z = −0.153, p = 0.878*); Spec-Pax assembly (*Z = −0.941, p = 0.347*), disassembly (*Z = −1.580, p = 0.114*); FL-Vinc assembly (*Z = −0.561, p = 0.575*), disassembly (*Z = −0.255, p = 0.799*); or Spec-Vinc assembly (*Z = −1.122, p = 0.262*), disassembly (*Z = −1.172, p = 0.241*). For panels A and D, Bar, 1 μm.

**Figure 4:**
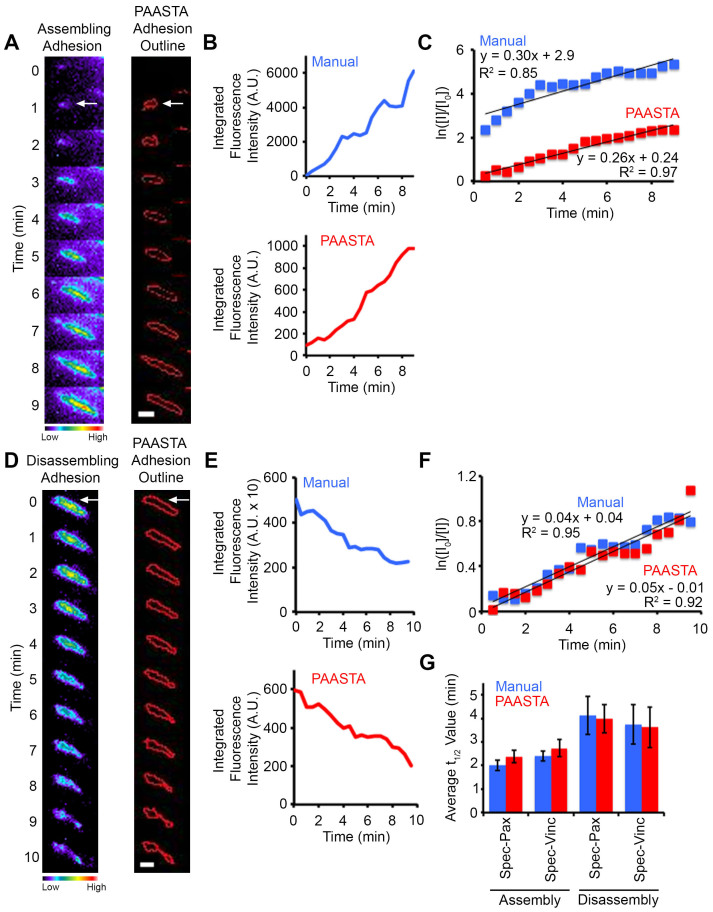
Manual and PAASTA analysis of adhesion assembly and disassembly in U2OS cells. (A,D) Left, Montages of time-lapse TIRF images for an assembling (panel A) and disassembling (panel D) adhesion (white arrows) in cells expressing GFP-paxillin are shown in pseudo-color coding, which indicates the range of fluorescence intensities. Cool colors correspond to lower intensity values, and warm colors correspond to higher intensity values. Right, Outlines of the adhesions as segmented by PAASTA. (B,E) Graphs of the fluorescence intensities of the assembling (panel B) and disassembling (panel E) adhesion are shown for both Manual and PAASTA tracking. (C,F) Plots of the natural log of the fluorescence intensity of the adhesions at given time points (I) relative to the initial fluorescence intensity (I_0_) are shown. Trendlines with the corresponding equation (y = mx + b) and R^2^ values are shown for fluorescence intensities that were obtained manually (Manual) and with PAASTA. The slopes of these graphs (m) are the apparent rate constant for adhesion assembly (panel C) or the rate constant for adhesion disassembly (panel F). (G) The average t_1/2_ values for adhesion assembly and disassembly for cells expressing Spec-Pax or Spec-Vinc are shown for Manual and PAASTA adhesion tracking. S.E.M. was calculated from: 20–21 adhesions (10–11 assembly, 10 disassembly) for each construct. A total of 14 cells were analyzed for adhesion assembly and disassembly. A Wilcoxon rank sum test showed no statistically significant difference between Manual and PAASTA tracking for Spec-Pax assembly (*Z = −1.682, p = 0.093*), disassembly (*Z = −0.153, p = 0.878*); or Spec-Vinc assembly (*Z = −1.112, p = 0.266*), disassembly (*Z = −0.255, p = 0.799*). For panels A and D, Bar, 1 μm.

We manually validated the automated tracking data received from PAASTA from time-series of cells expressing the fluorescently-tagged adhesion proteins, paxillin or vinculin. In these experiments, GFP-paxillin transfected HT1080 cells were plated on glass bottom dishes, which were coated with the ECM protein fibronectin (2D substrate), and imaged using total internal reflection fluorescence (TIRF) microscopy (Supplementary Movie 1). From these images, we manually tracked individual adhesions, measured fluorescence intensities in these adhesions, and quantified the kinetics of adhesion assembly and disassembly as previously described^9^. We next compared this manual adhesion data to the output generated by PAASTA using the same raw images. The relative changes in fluorescence intensities obtained from PAASTA for assembling adhesions was similar to that measured manually (Fig. 3A and B). To calculate apparent rate constants for adhesion assembly, we generated semilogarithmic plots of fluorescence intensities of individual adhesions as a function of time. The slopes of these graphs, which correspond to the apparent rate constant for adhesion assembly, were similar for the manually generated data and the data from PAASTA (Fig. 3C). In addition, for disassembling adhesions, the fluorescence intensity profiles attained manually and from PAASTA were similar (Fig. 3D and E), and the rate constants for adhesion disassembly were comparable for data obtained manually and with PAASTA (Fig. 3F). Indeed, the rate constants for adhesion assembly and disassembly, which we express as t_1/2_ values, that were obtained from manually tracking GFP-paxillin adhesions were very similar to those attained with PAASTA (Fig. 3G). An individual value plot shows the range of t_1/2_ values for adhesion assembly and disassembly (Supplementary Fig. S1). Moreover, in HT1080 cells expressing GFP-vinculin, another adhesion protein, the t_1/2_ values for adhesion assembly and disassembly were comparable for manual adhesion tracking and PAASTA (Fig. 3H). When we extended these observations to U2OS cells, we obtained very similar results for adhesion assembly (Fig. 4A–C and Supplementary Movie 2) and adhesion disassembly (Fig. 4D–F and Supplementary Movie 2). Thus, these results indicate that PAASTA accurately tracks and analyzes adhesion dynamics.

### Applications of PAASTA to adhesion dynamics

**Figure 5:**
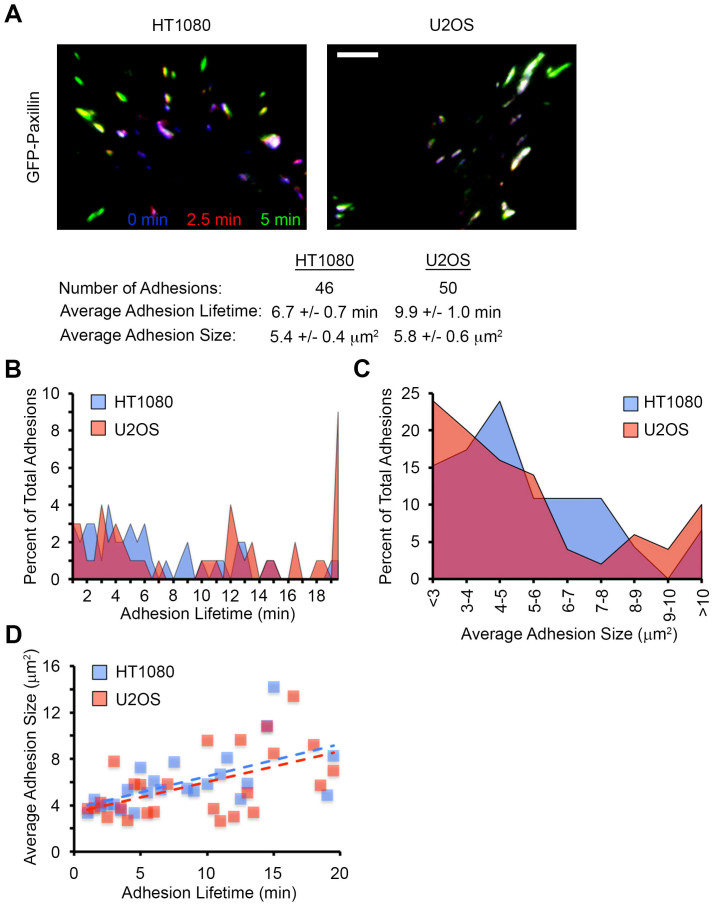
Capabilities of PAASTA for adhesion analysis. (A) A three-color temporal overlay is shown for an HT1080 and U2OS cell expressing GFP-paxillin. In general, blue and purple adhesions correspond to disassembly, green and yellow adhesions indicate assembly, and white adhesions are stable. The total number of adhesions, average adhesion lifetime, and average adhesion size calculated for these cells using PAASTA is shown below. Bar, 5 μm. (B) The percent of total adhesions with a given lifetime is shown for both cells in panel A. (C) The percent of total adhesions of a given size are shown for both cells in panel A. (D) The average adhesion size is plotted as a function of their lifetime for cells in panel A. Dashed lines represent the trendline for the indicated cell.

To further demonstrate the capabilities of PAASTA, we compared adhesions in HT1080 and U2OS cells (Fig. 5A). In these time-series, PAASTA tracked a total of 46 adhesions in the HT1080 cell and 50 adhesions in the U2OS cell. The average adhesion lifetime, defined as the total time an adhesion was observed during the time course, was 6.7 ± 0.7 min and 9.9 ± 1.0 min for the HT1080 and U2OS cell, respectively. The U2OS cell had more adhesions with a lifetime of greater than 19 min compared to the HT1080 cell (Fig. 5B). The average adhesion size for the HT1080 and U2OS cell was 5.4 ± 0.4 μm^2^ and 5.8 ± 0.6 μm^2^, respectively. Interestingly, even though the average size was comparable between the two cells, the U2OS cell had more small and large adhesions, while the HT1080 cell had a majority of moderately sized adhesions (Fig. 5C). Furthermore, the two cells showed very similar trends when comparing adhesion lifetime to adhesion size (Fig. 5D).

### Analysis of adhesion dynamics in a 3D environment with PAASTA

An attractive feature of PAASTA is that it is designed to analyze adhesion dynamics in 3D environments. *Kubow et al.*^20^ recently showed that very low expression of GFP-tagged adhesion proteins under the control of a truncated CMV promoter is ideal for imaging adhesions in 3D matrices. Hence, we employed this approach to generate time-lapse images for analysis of adhesions with PAASTA. In initial experiments, we expressed GFP-paxillin and GFP-vinculin cDNAs with the truncated CMV promoter (Spec-paxillin and Spec-vinculin) in both HT1080 and U2OS cells. We subsequently imaged these cells using time-lapse microscopy and quantified the t_1/2_ values for adhesion assembly and disassembly both manually and with PAASTA. The average t_1/2_ values obtained with these truncation constructs were quite similar to those attained in HT1080 cells with GFP-paxillin and GFP-vinculin with the full-length CMV promoter (FL-paxillin and FL-vinculin) (Fig. 3G and H). Moreover, we observed comparable t_1/2_ values with these truncation constructs in U2OS cells (Fig. 4G). We therefore proceeded to use these constructs to analyze adhesion assembly and disassembly in U2OS cells embedded in 3D type I collagen matrices.

**Figure 6:**
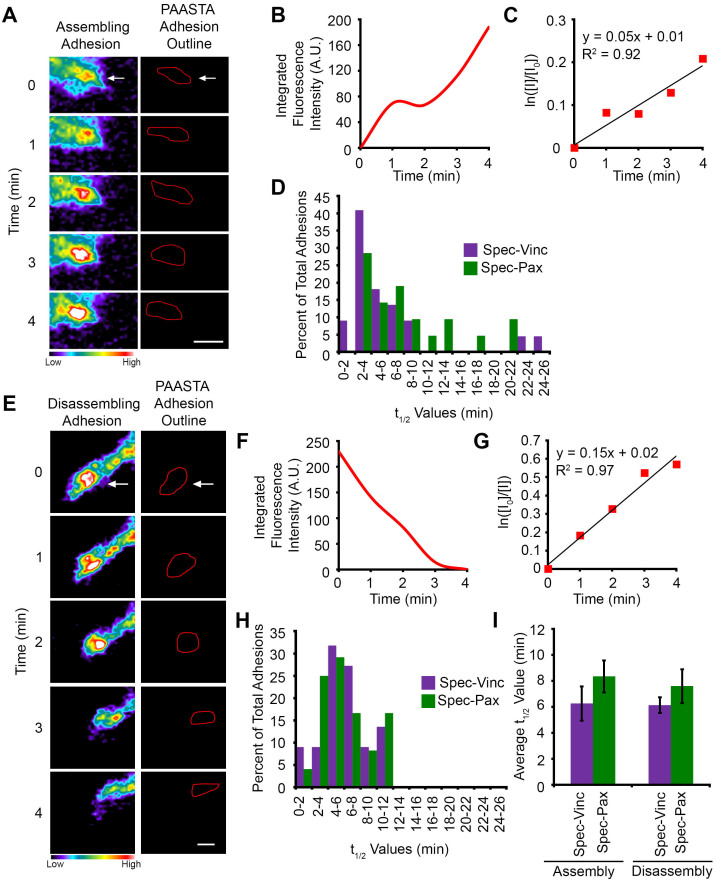
Analysis of adhesion dynamics for U2OS cells embedded in 3D type I collagen matrices using PAASTA. (A,E) Left, Time-lapse images of an assembling (panel A) and a disassembling (panel E) adhesion (white arrows) in Spec-Pax expressing cells embedded in a 3D type I collagen matrix are shown. The images are shown in pseudo-color coding to indicate the range of fluorescence intensities. Warm colors represent higher fluorescence intensities while cool colors denote lower fluorescence intensities. Right, Outlines of the adhesions as segmented by PAASTA. (B,F) Graphs of the fluorescence intensities of the assembling (panel B) and disassembling (panel F) adhesion are shown for PAASTA adhesion tracking. (C,G) Plots of the natural log of the fluorescence intensity of the adhesions at given time points (I) relative to the initial fluorescence intensity (I_0_) are shown. Trendlines with the corresponding equation (y = mx + b) and R^2^ values are shown. The slopes of these graphs (m) are the apparent rate constant for adhesion assembly (panel C) or the rate constant for adhesion disassembly (panel G). (D,H) Histograms of the distribution of t_1/2_ values for adhesion assembly (panel D) and disassembly (panel H) are shown for cells expressing either Spec-Pax or Spec-Vinc. (I) The average t_1/2_ values for adhesion assembly and disassembly are shown for cells expressing either Spec-Pax or Spec-Vinc. S.E.M. was calculated from 47 adhesions (21 assembly, 26 disassembly) for Spec-Pax and 44 adhesions (22 assembly, 22 disassembly) for Spec-Vinc. A total of 9 cells were used for the analysis of adhesion assembly and disassembly. A Wilcoxon rank sum test showed no statistically significant difference between Spec-Pax and Spec-Vinc for assembly (*Z = −1.269, p = 0.205*) or disassembly (*Z = −0.406, p = 0.685*). For panels A and E, Bar, 1 μm.

We generated multidimensional time-lapse images (with a z-interval of 0.5 μm) for U2OS cells expressing either Spec-paxillin or Spec-vinculin (Supplementary Movie 3). Only cells that were at least 100 μm from the coverslips were imaged to ensure they were embedded in the 3D matrices. Adhesions were identified in each z-plane and tracked as a function of time through the z-stack using the nearest neighbor algorithm. This approach allows adhesions that are moving through different focal planes to be tracked over time. Adhesion ID numbers were exported along with the average integrated fluorescence intensities from the z-planes in which the adhesions were present. A profile of the fluorescent intensities obtained from PAASTA showed an adhesion assembling and disassembling in 3D type I collagen matrices (Supplementary Fig. S2A). Changes in fluorescence intensities for an assembling (Fig. 6A and B) and disassembling (Fig. 6E and F) adhesion are also shown along with semilogarithmic plots of fluorescence intensities over time (Fig. 6C and G). The average R^2^ values for the adhesion assembly and disassembly plots are 0.89 ± 0.01 (S.E.M. from 43 adhesions) and 0.88 ± 0.01 (S.E.M. from 48 adhesions), respectively. From these plots, t_1/2_ values were calculated for assembly and disassembly of adhesions tracked through PAASTA (Fig. 6I). An individual value plot shows the range of t_1/2_ values for adhesion assembly and disassembly (Supplementary Fig. S2B). Distribution plots revealed that most paxillin and vinculin-containing adhesions have t_1/2_ values of less than 10 min for assembly and disassembly in 3D type I collagen matrices (Fig. 6D and H). Moreover, the average adhesion lifetime was 13.4 ± 1.0 min, and the average adhesion size was 6.1 ± 0.4 μm^2^ for U2OS cells in 3D type I collagen matrices (Supplementary Fig. S3A and B). However, these plots show some variability with some adhesions having t_1/2_ values of greater than 20 min, lifetimes of 30 min, and average sizes larger than 10 μm^2^. Others have similarly reported variability in adhesion parameters, including their size, distribution, shape, and location^31^,^32^,^33^. Interestingly, the average adhesion size correlated with the adhesion lifetime (Supplementary Fig. S3C), suggesting that smaller adhesions have a shorter lifetime than the larger adhesions. Taken together, our data demonstrate that PAASTA is a useful platform for rapidly analyzing multiple adhesion parameters in 3D environments over time.

## Discussion

Since adhesions were first shown to be direct regions of contact between a cell and the substratum using interference reflection microscopy, they have been extensively studied, characterized, and analyzed on flat 2D substrates^34^,^35^,^36^. These studies have been extremely beneficial in understanding adhesion organization, regulation, and structure and have laid the foundation for the identification of adhesions in more complex, physiologic 3D environments^17^,^26^,^37^,^38^. Adhesions in 3D environments differ from adhesions on 2D substrates in some respects and appear to more closely resemble adhesions *in vivo*^17^,^21^,^22^. These observations warrant a more thorough analysis of adhesion organization, regulation, and dynamics in 3D environments. However, current analysis methods and systems for quantifying adhesion parameters, such as assembly, disassembly, and size, in cells plated on 2D substrates^9^,^39^,^40^ have not been shown to have the capability to analyze adhesion dynamics in 3D environments. This lack of available tools to analyze adhesions in 3D environments has hindered progress toward understanding adhesions in 3D. Consequently, we have developed a reliable, powerful platform (PAASTA) for the large-scale, rapid analysis of adhesions in 3D environments. PAASTA uses multidimensional images to identify and track adhesions through z-planes over time, permitting adhesion dynamics to be quantified in 3D. Therefore, PAASTA should prove to be a useful tool for investigating adhesions in 3D environments and for deciphering the molecular mechanisms that regulate adhesion dynamics in 3D.

Using PAASTA, we examined adhesion assembly and disassembly in U2OS cells embedded in 3D type I collagen matrices. The t_1/2_ values for adhesion assembly and disassembly were approximately 7 min (Fig. 6). Based on data obtained on 2D substrates^41^,^42^, these results suggest that adhesions in cells in 3D type I collagen matrices are relatively stable. Small adhesions in cells on 2D substrates have been reported to turn over in a few minutes (Fig. 3)^41^,^42^,^43^. However, adhesion turnover on 2D substrates could differ among cell types, ECM proteins, and ECM concentration. Furthermore, adhesion in 3D is most likely more complex than adhesion on 2D substrates and influenced by factors, such as matrix composition, pliability, pore size, fiber alignment, as well as the immediate microenvironment of each adhesion^21^. Indeed, fiber orientation was shown to modulate adhesion size and maturation in 3D type I collagen matrices^28^. Therefore, future studies are needed to understand adhesion dynamics in 3D environments and how they compare to 2D substrates.

Investigating adhesions in 3D environments is attractive because they more closely resemble adhesions *in vivo* compared to adhesions on 2D substrates^17^,^22^. Thus, studies characterizing adhesions in 3D matrices will provide a wealth of information on the behavior of adhesions in more physiologic environments. These studies could also serve as a foundation for examining adhesions *in vivo*, which is currently difficult with available technologies. As innovative methodologies emerge, new analytical tools will be needed to characterize adhesions in 3D environments as well as *in vivo*.

We used PAASTA to analyze adhesion turnover, lifetime, and size in 3D matrices; however, PAASTA is a versatile, automated platform for analyzing many different adhesion characteristics in 3D environments and *in vivo*. For example, future incarnations of this platform could include features that would assess adhesion shape, distribution, and distance from the cell edge. Because PAASTA is built on the CellAnimation MATLAB platform, other modules can easily be added to the workflow. As technology advances, and adhesions can be more readily visualized *in vivo*, PAASTA should also provide a valuable platform for analyzing these structures.

## Methods

### Cell culture and transfection

HT1080 and U2OS cells were maintained in Dulbeco's Modified Eagles Medium (DMEM) (Invitrogen, Carlsbad, CA) that was supplemented with 10% fetal bovine serum (FBS) (HyClone, Logan, UT) and 1% penicillin/streptomycin (Invitrogen). Both HT1080 and U2OS cells were transiently transfected with Lipofectamine™ 2000 (Invitrogen) according to the manufacturer's instructions.

### Imaging adhesions on 2D substrates

Cells transfected with either GFP-paxillin, GFP-vinculin, Spec-paxillin, or Spec-vinculin were plated on glass-bottomed dishes, which were precoated with 2.5 μg/mL fibronectin (Sigma, St. Louis, MO), and permitted to adhere for 1 h at 37^o^C. While imaging, cells were maintained in SFM4MAb™ media (Hyclone) supplemented with 2% FBS, pH 7.4. Cells were imaged on an inverted Olympus IX71 microscope (Melville, NY), which was equipped with a Retiga EXi CCD camera (QImaging, Surrey, BC) and an Olympus PlanApo 60× OTIRFM objective (NA 1.45), using MetaMorph software (Molecular Devices, Sunnyvale, CA). TIRF images were acquired by exciting with a 488 nm laser line from an Argon-Ion laser (Prairie Technologies, Middleton, WI). For TIRF imaging, a z488/543 rpc filter was used (Chroma, Brattleboro, VT). GFP-vinculin was a kind gift from Susan Craig (Johns Hopkins University, Baltimore, MD). Spec-paxillin and Spec-vinculin were generously provided by Rick Horwitz (University of Virginia, Charlottesville, VA).

### Imaging adhesions in 3D matrices

Rat-tail type I collagen (BD Biosciences, Bedford, MA) was mixed with sterile 10× DMEM (Invitrogen), sterile dH_2_O, FBS, and 1N NaOH to a final concentration of 2 mg/mL type I collagen, 10% FBS and 1× DMEM. NaOH was used for neutralization at 0.023 mL × the volume of type I collagen solution. U2OS cells transfected with either Spec-paxillin or Spec-vinculin were seeded (~1.5 × 10^5^ cells) into 300 μL of type I collagen solution and pipetted into the bottom of glass-bottomed dishes. The type I collagen solution with embedded cells was allowed to polymerize for at least 30 min at 37°C in a cell culture incubator with 5% CO_2_. Subsequently, 2 mL of culture medium was gently added to each dish, and cells were incubated for approximately 18 h at 37°C in a cell culture incubator with 5% CO_2_. Prior to imaging, the culture medium was replaced with SFM4MAb™ medium supplemented with 10% FBS, pH 7.4.

Z-series were acquired using a Quorum WaveFX-X1 spinning disk confocal system with a Yokogawa CSU-X1 spinning disk (Yokogawa Electric Corporation, Newnan, GA) modified with a Borealis upgrade (Guelph, Canada) and a Nikon Eclipse Ti microscope that was equipped with an EM-CCD camera (Hamamatsu, Hamamatsu City, Japan) and a Plan Fluor 40× objective (N.A. 1.3). Z-series were collected using MetaMorph software at time intervals of 45 sec - 1 min with a z-interval of 0.5 μm. GFP was excited with a 491 nm laser line and imaged with a 525/50 emission filter (Semrock, Rochester, NY). Only cells that were completely embedded within the 3D collagen matrix (at least 100 μm from the coverslips) were imaged.

### Manual adhesion analysis

All manual image analysis was performed using MetaMorph software. Individual adhesions were identified, and a region was created with the trace region tool that completely outlined the adhesion at the timepoint in which the adhesion had the greatest area. The integrated intensity for the fluorescently-tagged adhesion marker (paxillin or vinculin) in this region was recorded over time. An exact duplicate region was positioned within an area adjacent to the tracked adhesion, which was inside the cell and did not contain an adhesion at any timepoint. The integrated fluroescence intensity of this region was then used as background and was subtracted from each timepoint from the region containing the adhesion. These background-corrected data were then used in further processing steps to calculate adhesion kinetics.

### Adhesion analysis with PAASTA

For adhesion analysis, a Gaussian smoothing module was applied to the raw images to reduce noise and then corrected for uneven background illumination by dividing each smoothed image with a low pass filtered version of itself. A local thresholding module was used to compare the intensity of each pixel with the mean value of the local neighborhood of the pixel in order to detect adhesions. Cell outlines were detected by thresholding the background-corrected images using a global intensity threshold module. Adhesions were selected by combining the binary mask of the cell with the binary image of the adhesions. Kernel sizes and thresholds were user designated because images can vary due to experimental conditions. Individual adhesions, which were assigned ID numbers, were tracked over time using a nearest neighbor algorithm. Adhesions that split into two adhesions during imaging were tracked as separate adhesions; the adhesion that remained closest to the previous image was tracked with the same ID number whereas the other adhesion was assigned a new ID number. Adhesion ID numbers, individual adhesion integrated intensities, and area information at every time point were exported to comma-separated text files for further analysis. A set of images showing the detected adhesion outlines, with or without ID numbers, were overlaid on the original images for manual validation of the automated quantification. Adhesions were imaged with a temporal resolution (≤1 min) that was sufficient to obtain numerous data points for adhesion analysis. Furthermore, because the temporal resolution was high, the number of adhesions did not vary greatly between subsequent images, which allowed the user to adjust any ID numbers due to splitting and merging adhesions. The vast majority of adhesions in cells were correctly identified and tracked using PAASTA, indicating that the adhesion density was amenable to tracking with the nearest neighbor algorithm. A modified nearest neighbor algorithm or binary integer programming can be added to PAASTA if the adhesion density increases dramatically and the nearest neighbor algorithm is no longer sufficient for adhesion tracking.

### Calculating rate constants for adhesion assembly and disassembly

The background-corrected integrated fluorescence intensities in individual adhesions were determined manually or with PAASTA. Semilogarithmic plots of the background-subtracted fluorescence intensities over time were then generated as follows: *ln [I_0_/I]* vs. time for adhesion disassembly and *ln [I/I_0_]* vs. time for adhesion assembly, where *[I]* is the intensity of the adhesion at a given timepoint and *[I_0_]* is the initial intensity of the adhesion. Data were then fitted with a linear trendline, and rate constants were determined from the slopes. Rate constants were used to calculate half-life values for adhesion assembly and disassembly using the equation: *t_1/2_ = ln(2)/k*, where k is the rate constant.

## Author Contributions

J.A.B. and D.J.W. designed the experiments. J.A.B. performed the experiments. J.A.B. and N.L.D. analyzed the data. S.H., W.G. and V.Q. created the automated platform PAASTA and provided scientific input and manuscript suggestions. J.A.B., N.L.D. and D.J.W. wrote the paper.

## Supplementary Material

Supplementary Information Movie S1

Supplementary Information Movie S2

Supplementary Information Movie S3

Supplementary Information Supplementary Information

## References

[b1] Hynes R. O. Integrins: bidirectional, allosteric signaling machines. Cell 110, 673–687 (2002). 12297042 10.1016/s0092-8674(02)00971-6

[b2] Burridge K. & Chrzanowska-Wodnicka M. Focal adhesions, contractility, and signaling. Annu Rev Cell Dev Biol 12, 463–518, 10.1146/annurev.cellbio.12.1.463 (1996). 8970735

[b3] Kanchanawong P. *et al.* Nanoscale architecture of integrin-based cell adhesions. Nature 468, 580–584, 10.1038/nature09621 (2010). 21107430 PMC3046339

[b4] Berrier A. L. & Yamada K. M. Cell-matrix adhesion. J Cell Physiol 213, 565–573, 10.1002/jcp.21237 (2007). 17680633

[b5] Geiger B., Bershadsky A., Pankov R. & Yamada K. M. Transmembrane crosstalk between the extracellular matrix--cytoskeleton crosstalk. Nat Rev Mol Cell Biol 2, 793–805, 10.1038/35099066 (2001). 11715046

[b6] Dubash A. D. *et al.* Chapter 1. Focal adhesions: new angles on an old structure. Int Rev Cell Mol Biol 277, 1–65, 10.1016/S1937-6448(09)77001-7 (2009). 19766966

[b7] Reddig P. J. & Juliano R. L. Clinging to life: cell to matrix adhesion and cell survival. Cancer Metastasis Rev 24, 425–439, 10.1007/s10555-005-5134-3 (2005). 16258730

[b8] Wolfenson H., Lavelin I. & Geiger B. Dynamic regulation of the structure and functions of integrin adhesions. Dev Cell 24, 447–458, 10.1016/j.devcel.2013.02.012 (2013). 23484852 PMC3878073

[b9] Webb D. J. *et al.* FAK-Src signalling through paxillin, ERK and MLCK regulates adhesion disassembly. Nat Cell Biol 6, 154–161, 10.1038/ncb1094 (2004). 14743221

[b10] Wehrle-Haller B. Assembly and disassembly of cell matrix adhesions. Curr Opin Cell Biol 24, 569–581, 10.1016/j.ceb.2012.06.010 (2012). 22819514

[b11] Vicente-Manzanares M. & Horwitz A. R. Adhesion dynamics at a glance. J Cell Sci 124, 3923–3927, 10.1242/jcs.095653 (2011). 22194302 PMC3244977

[b12] Geiger B. & Yamada K. M. Molecular architecture and function of matrix adhesions. Cold Spring Harb Perspect Biol 3, 10.1101/cshperspect.a005033 (2011). PMC3101841 21441590

[b13] Miyamoto S. *et al.* Integrin function: molecular hierarchies of cytoskeletal and signaling molecules. J. Cell Biol. 131, 791–805 (1995). 7593197 10.1083/jcb.131.3.791 PMC2120620

[b14] Zaidel-Bar R., Ballestrem C., Kam Z. & Geiger B. Early molecular events in the assembly of matrix adhesions at the leading edge of migrating cells. J. Cell Sci. 116, 4605–4613 (2003). 14576354 10.1242/jcs.00792

[b15] Burridge K., Fath K., Kelly T., Nuckolls G. & Turner C. Focal adhesions: transmembrane junctions between the extracellular matrix and the cytoskeleton. Annu Rev Cell Biol 4, 487–525, 10.1146/annurev.cb.04.110188.002415 (1988). 3058164

[b16] Hanein D. & Horwitz A. R. The structure of cell-matrix adhesions: the new frontier. Curr Opin Cell Biol 24, 134–140, 10.1016/j.ceb.2011.12.001 (2012). 22196929 PMC3294145

[b17] Cukierman E., Pankov R., Stevens D. R. & Yamada K. M. Taking cell-matrix adhesions to the third dimension. Science 294, 1708–1712, 10.1126/science.1064829 (2001). 11721053

[b18] Petroll W. M. & Ma L. Direct, dynamic assessment of cell-matrix interactions inside fibrillar collagen lattices. Cell Motil Cytoskeleton 55, 254–264, 10.1002/cm.10126 (2003). 12845599

[b19] Deakin N. O. & Turner C. E. Distinct roles for paxillin and Hic-5 in regulating breast cancer cell morphology, invasion, and metastasis. Mol Biol Cell 22, 327–341, 10.1091/mbc.E10-09-0790 (2011). 21148292 PMC3031464

[b20] Kubow K. E. & Horwitz A. R. Reducing background fluorescence reveals adhesions in 3D matrices. Nat Cell Biol 13, 3–5, 10.1038/ncb0111-3 (2011). 21173800 PMC3083631

[b21] Harunaga J. S. & Yamada K. M. Cell-matrix adhesions in 3D. Matrix Biol 30, 363–368, 10.1016/j.matbio.2011.06.001 (2011). 21723391 PMC3191245

[b22] Geraldo S. *et al.* Do cancer cells have distinct adhesions in 3D collagen matrices and in vivo? Eur J Cell Biol 91, 930–937, 10.1016/j.ejcb.2012.07.005 (2012). 22939225

[b23] Cukierman E., Pankov R. & Yamada K. M. Cell interactions with three-dimensional matrices. Curr Opin Cell Biol 14, 633–639 (2002). 12231360 10.1016/s0955-0674(02)00364-2

[b24] Li S. *et al.* Genomic analysis of smooth muscle cells in 3-dimensional collagen matrix. FASEB J 17, 97–99, 10.1096/fj.02-0256fje (2003). 12475912

[b25] Hakkinen K. M., Harunaga J. S., Doyle A. D. & Yamada K. M. Direct comparisons of the morphology, migration, cell adhesions, and actin cytoskeleton of fibroblasts in four different three-dimensional extracellular matrices. Tissue Eng Part A 17, 713–724, 10.1089/ten.TEA.2010.0273 (2011). 20929283 PMC3043991

[b26] Tamariz E. & Grinnell F. Modulation of fibroblast morphology and adhesion during collagen matrix remodeling. Mol Biol Cell 13, 3915–3929, 10.1091/mbc.E02-05-0291 (2002). 12429835 PMC133603

[b27] Doyle A. D. *et al.* Micro-environmental control of cell migration--myosin IIA is required for efficient migration in fibrillar environments through control of cell adhesion dynamics. J Cell Sci 125, 2244–2256, 10.1242/jcs.098806 (2012). 22328520 PMC3367941

[b28] Kubow K. E., Conrad S. K. & Horwitz A. R. Matrix microarchitecture and myosin II determine adhesion in 3D matrices. Curr Biol 23, 1607–1619, 10.1016/j.cub.2013.06.053 (2013). 23932405 PMC3773288

[b29] Doyle A. D., Wang F. W., Matsumoto K. & Yamada K. M. One-dimensional topography underlies three-dimensional fibrillar cell migration. J Cell Biol 184, 481–490, 10.1083/jcb.200810041 (2009). 19221195 PMC2654121

[b30] Georgescu W., Wikswo J. P. & Quaranta V. CellAnimation: an open source MATLAB framework for microscopy assays. Bioinformatics 28, 138–139, 10.1093/bioinformatics/btr633 (2012). 22121157 PMC3244774

[b31] Welf E. S., Ogunnaike B. A. & Naik U. P. Quantitative statistical description of integrin clusters in adherent cells. IET Syst Biol 3, 307–316, 10.1049/iet-syb.2009.0009 (2009). 21028922

[b32] Welf E. S., Naik U. P. & Ogunnaike B. A. Probabilistic modeling and analysis of the effects of extra-cellular matrix density on the sizes, shapes, and locations of integrin clusters in adherent cells. BMC Biophys 4, 15, 10.1186/2046-1682-4-15 (2011). 21827670 PMC3179437

[b33] Chien F. C., Kuo C. W., Yang Z. H., Chueh D. Y. & Chen P. Exploring the formation of focal adhesions on patterned surfaces using super-resolution imaging. Small 7, 2906–2913, 10.1002/smll.201100753 (2011). 21861294

[b34] Izzard C. S. & Lochner L. R. Cell-to-substrate contacts in living fibroblasts: an interference reflexion study with an evaluation of the technique. J Cell Sci 21, 129–159 (1976). 932106 10.1242/jcs.21.1.129

[b35] Heath J. P. & Dunn G. A. Cell to substratum contacts of chick fibroblasts and their relation to the microfilament system. A correlated interference-reflexion and high-voltage electron-microscope study. J Cell Sci 29, 197–212 (1978). 564353 10.1242/jcs.29.1.197

[b36] Horwitz A. R. The origins of the molecular era of adhesion research. Nat Rev Mol Cell Biol 13, 805–811, 10.1038/nrm3473 (2012). 23151664 PMC3692278

[b37] Vaughan M. B., Howard E. W. & Tomasek J. J. Transforming growth factor-beta1 promotes the morphological and functional differentiation of the myofibroblast. Exp Cell Res 257, 180–189, 10.1006/excr.2000.4869 (2000). 10854066

[b38] Wolf K. *et al.* Compensation mechanism in tumor cell migration: mesenchymal-amoeboid transition after blocking of pericellular proteolysis. J Cell Biol 160, 267–277, 10.1083/jcb.200209006 (2003). 12527751 PMC2172637

[b39] Berginski M. E., Vitriol E. A., Hahn K. M. & Gomez S. M. High-resolution quantification of focal adhesion spatiotemporal dynamics in living cells. PLoS One 6, e22025, 10.1371/journal.pone.0022025 (2011). 21779367 PMC3136503

[b40] Wurflinger T., Gamper I., Aach T. & Sechi A. S. Automated segmentation and tracking for large-scale analysis of focal adhesion dynamics. J Microsc 241, 37–53, 10.1111/j.1365-2818.2010.03404.x (2011). 21118203

[b41] Jean L. *et al.* Activation of Rac by Asef2 promotes myosin II-dependent contractility to inhibit cell migration on type I collagen. J Cell Sci 126, 5585–5597, 10.1242/jcs.131060 (2013). 24144700 PMC3860307

[b42] Broussard J. A. *et al.* The endosomal adaptor protein APPL1 impairs the turnover of leading edge adhesions to regulate cell migration. Mol Biol Cell 23, 1486–1499, 10.1091/mbc.E11-02-0124 (2012). 22379109 PMC3327316

[b43] Nayal A. *et al.* Paxillin phosphorylation at Ser273 localizes a GIT1-PIX-PAK complex and regulates adhesion and protrusion dynamics. J Cell Biol 173, 587–589, 10.1083/jcb.200509075 (2006). 16717130 PMC2063867

