# Supplementary material for: Automated Analysis of Cell-Matrix Adhesions in 2D and 3D Environments
Source: Sci Rep. 2015 Jan 29;5:8124. doi: 10.1038/srep08124 (PMC4309964; doi:10.1038/srep08124)
Supplement: Supplementary Information [file srep08124-s4.pdf]

Supplementary Information

Automated Analysis of Cell-Matrix Adhesions in 2D and 3D Environments

Joshua A. Broussard[1,‡], Nicole L. Diggins[1,‡], Stephen Hummel[2] , Walter Georgescu[2,3,4], Vito Quaranta[2,3,5], and Donna J Webb[1,3,5,*]

[1]Department of Biological Sciences and Vanderbilt Kennedy Center for Research on Human Development, [2]Center for Cancer Systems Biology at Vanderbilt, [3]Vanderbilt Institute for Integrative Biosystems Research and Education (VIBRE), [4]Department of Biomedical Engineering, and [5]Department of Cancer Biology, Vanderbilt University, Nashville, Tennessee 37235.

‡These authors have contributed to an equal extent.

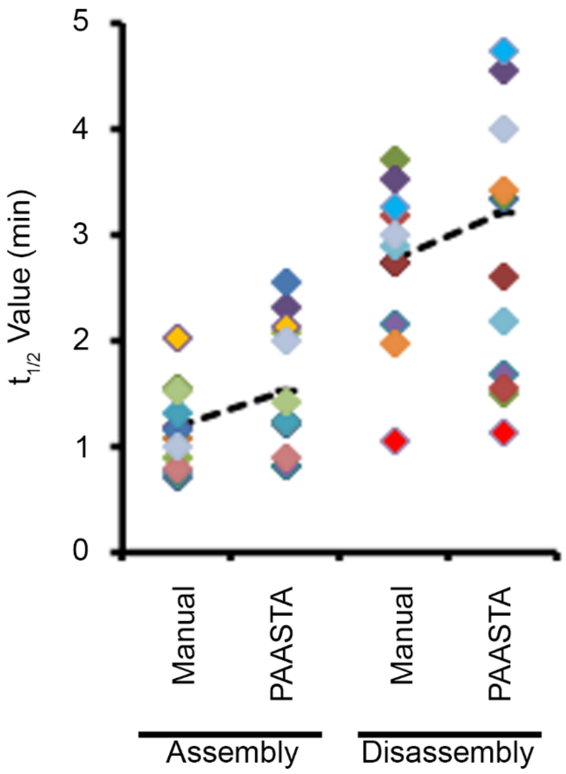

Figure S1. An individual value plot of $t_{1/2}$ values for adhesion assembly and disassembly. Individual $t_{1/2}$ values were obtained from TIRF imaging of HT1080 cells expressing GFP-paxillin with a truncated CMV promoter (Spec-Pax) with both manual and PAASTA tracking. Matching colors correspond to the same adhesion, and the dashed lines correspond to the average of each category.

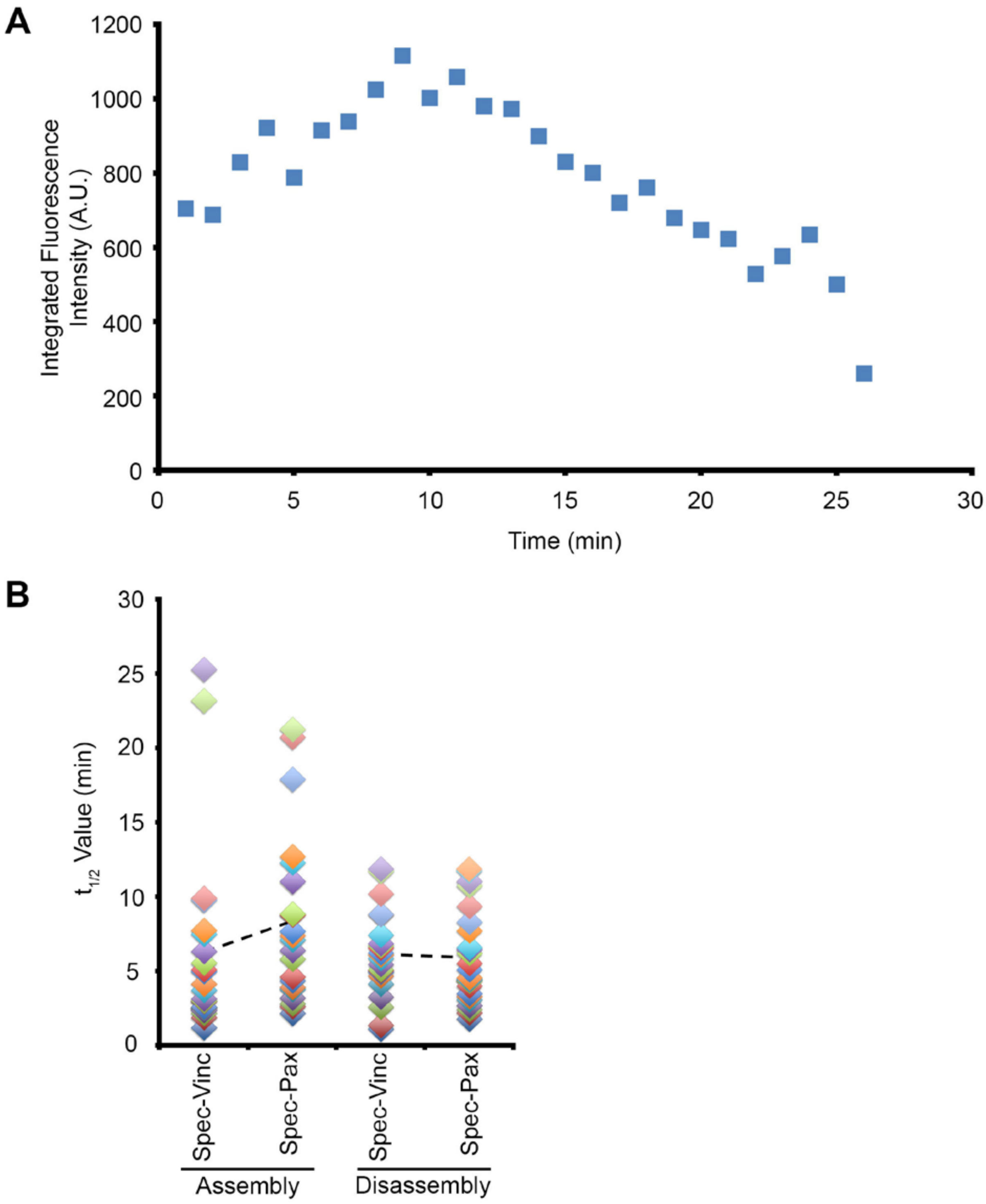

Figure S2. PAASTA tracking and analysis of adhesion assembly and disassembly in U2OS cells embedded in 3D matrices. (A) The integrated fluorescence intensites of an individual adhesion from a Spec-Pax expressing U2OS cell, embedded in a 3D type I collagen matrix, were attained using PAASTA and plotted as a function of time. The plot shows the adhesion assembling and disassembling over time. (B) Individual $t_{1/2}$ values were obtained from imaging U2OS cells, expressing Spec-Pax or Spec-Vinc, embedded in 3D type I collagen matrices. Dashed lines correspond to the average of each category.

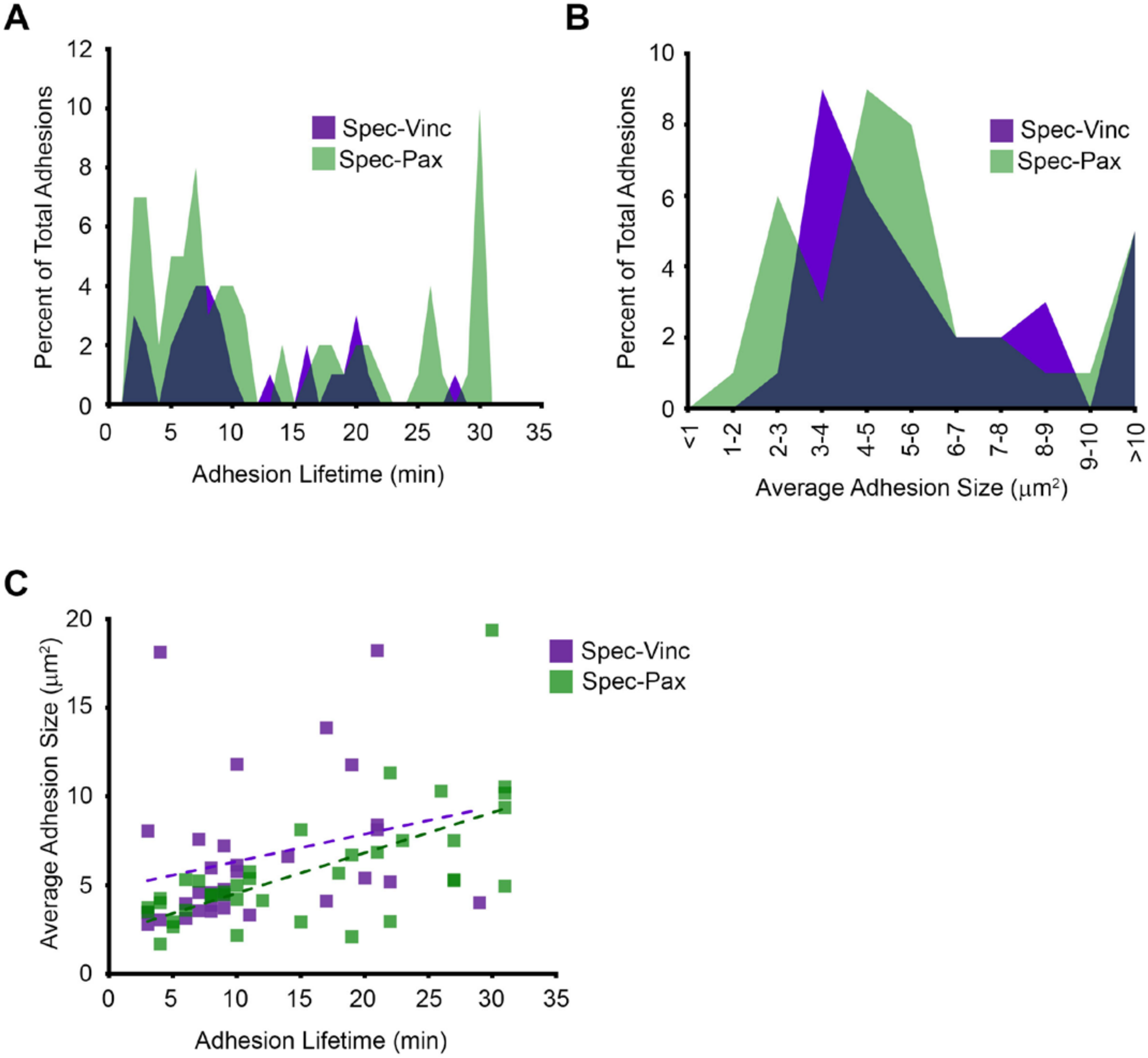

Figure S3. PAASTA analysis of adhesion parameters in U2OS cells embedded 3D matrices. Spec-Pax or Spec-Vinc-containing adhesions in U20S cells, embedded in 3D type I collagen matrices, were tracked using PAASTA, and the adhesion lifetime and average adhesion size were calculated. (A) The percent of total adhesions with a given lifetime is shown. (B) The percent of total adhesions of a given size are shown. (C) The average adhesion size is plotted as a function of their lifetime. Dashed lines represent the trendline for cells expressing the indicated constructs.

Movie S1. Adhesion assembly and disassembly on 2D fibronectin in HT1080 cells. HT1080 cells expressing GFP-paxillin were plated on 2D fibronectin and imaged using TIRF microscopy. This movie shows adhesions assembling and disassembling as a HT1080 cell protrudes. Images were acquired every 30 sec for 20 min. The frame rate for the movie is 15 frames/sec.

Movie S2. Assembly and disassembly of adhesions on 2D fibronectin in U2OS cells. U2OS cells expressing GFP-paxillin were plated on 2D fibronectin and imaged using TIRF microscopy. This movie demonstrates adhesions assembling and disassembling in a protrusion. Images were collected every 30 sec for 20 min. The frame rate for the movie is 15 frames/sec.

Movie S3. Assembly and disassembly of adhesions in 3D type I collagen matrices in U2OS cells. U2OS cells expressing GFP-tagged paxillin with a truncated CMV promoter (Spec-paxillin) were embedded in a 3D type I collagen matrix and imaged using spinning disk confocal microscopy. This movie shows an extending protrusion in which adhesions are assembling and disassembling (at the tip of the protrusion). Images were acquired every 1 min, and 15 time-lapse images were used to generate this movie. The frame rate for the movie is 15 frames/sec.
